# Draft Genome Sequence of the Mycoparasitic Oomycete *Pythium periplocum* Strain CBS 532.74

**DOI:** 10.1128/genomeA.00057-17

**Published:** 2017-03-23

**Authors:** Sandeep K. Kushwaha, Ramesh R. Vetukuri, Laura J. Grenville-Briggs

**Affiliations:** a Department of Plant Breeding, Swedish University of Agricultural Sciences, Alnarp, Sweden; b National Bioinformatics Infrastructure Sweden (NBIS), Department of Biology, Lund University, Lund, Sweden; c Department of Plant Protection Biology, Swedish University of Agricultural Sciences, Alnarp, Sweden

## Abstract

The oomycete *Pythium periplocum* is an aggressive mycoparasite of a number of plant pathogenic fungi and oomycetes and therefore has potential as a biological control agent. Here, we report the first draft genome sequence of *P. periplocum*, which comprises 35.89 Mb. It contains 1,043 scaffolds and 14,399 predicted protein-coding genes.

## GENOME ANNOUNCEMENT

The oomycetes are a lineage of filamentous eukaryotes related to the heterokont (brown) algae. Many oomycetes are plant pathogens, but *Pythium periplocum* is directly parasitic to fungi and other oomycetes (1, 2). Given the negative environmental impact of synthetic pesticides, and the aggressive and adaptive behavior of fungal and oomycete plant pathogens, understanding the molecular determinants of *P. periplocum* mycoparasitism, through whole-genome sequencing, will aid in the development of more environmentally sustainable disease controls.

*P. periplocum* was cultured on V8 media, and DNA was extracted as described previously (3). Illumina HiSeq 150-bp paired-end sequencing was performed at SciLifeLab, Sweden. FastQC Trimmomatic tools (4) were used for quality assessment and control of the raw data; 40.18 Mb of trimmed reads were used for assembly. Best *k*-mer length was identified with KmerGenie (5). SPAdes version 3.5.0, a de Bruijn graph-based assembler, was used for *de novo* assembly (6); 35.89 Mb were assembled into 1,043 scaffolds of more than 2 kb in length (mean coverage, 105×). QUAST (7) was used to assess the quality of the assembly (*N*_50_: 95,690 bp; *N*_75_: 48,238 bp; *L*_50_: 105 bp; *L*_75_: 227 bp; longest scaffold: 732,016 bp; number of scaffolds >50 kb: 216; and number of scaffolds >10 kb: 498), and a 97.98% assembly completeness was estimated using CEGMA version 2.5 (8). The draft genome was compared to eukaryotic genome models using Augustus (9). It contains 14,399 predicted protein-coding genes greater than 100 amino acids in length. Pfam domains were assigned to 9,168 of these and secreted protein signals to 1,415; 1,853 significant matches to the CAZy database (10) (E-value: 1e^−10^) included glycoside hydrolases, glycosyltransferases, carbohydrate-binding modules, polysaccharide lyases, carbohydrate esterases, and redox enzymes.

### Accession number(s).

This whole-genome shotgun project has been deposited at DDBJ/ENA/GenBank under the accession number MRVE00000000. The version described in this paper is the first version, MRVE01000000.
